# Community readiness and acceptance for the implementation of a novel malaria vaccine among at-risk children in sub-saharan Africa: a systematic review protocol

**DOI:** 10.1186/s12936-024-04995-y

**Published:** 2024-06-10

**Authors:** Eustes Kigongo, Amir Kabunga, Marc Sam Opollo, Raymond Tumwesigye, Marvin Musinguzi, Anne Ruth Akello, Jannat Nabaziwa, Temesgen Geta Hardido, Sean Steven Puleh

**Affiliations:** 1 Faculty of Public Health, Lira University, Lira, Uganda; 2 Faculty of Medicine, Lira University, Lira, Uganda; 3 Faculty of Medicine, Mbarara University of Science and Technology, Mbarara, Uganda; 4 School of Nursing, Wolaita Sodo University, Wolaita, Soddo, Ethiopia

## Abstract

**Background:**

The World Health Organization novel malaria vaccine for at-risk children has the potential to greatly reduce the current malaria burden in sub-Saharan Africa. However, most studies have reported contradictory findings regarding community willingness for the vaccine, which could easily undermine the expected benefits of the vaccine. This study aims to ascertain the current state of community readiness and acceptance for the implementation of a novel malaria vaccine (RTS,S/ASO1) among at-risk children in sub-Saharan Africa, based on available evidence.

**Methods:**

This study will follow the Preferred Reporting Items for Systematic Reviews and Meta-analyses protocol (PRISMA-P) guidelines. Relevant studies will be comprehensively searched from PubMed, ScienceDirect, Web of Science, Google Scholar, and African journals online, in accordance with the Cochrane search guidelines. Two independent reviewers will screen titles, abstracts and full texts of eligible studies based on some specified eligibility criteria. When it is feasible to conduct a meta-analysis, a random effects model will be employed to estimate the common effect due to anticipated high heterogeneity of the data. The effect measure for readiness or acceptance will be reported as a pooled proportion with corresponding 95% confidence interval. Additionally, odds ratios with 95% confidence interval will be estimated to assess factors associated with readiness. These will be presented on a forest plot.

**Dissemination plans:**

The findings of the study will be peer-reviewed and published in a scientific journal. Conference presentations will also be made to the different stakeholders in the malaria vaccination campaigns.

**Systematic review registration:**

The protocol has been registered with PROSPERO Registration Number: CRD42023480528.

**Supplementary Information:**

The online version contains supplementary material available at 10.1186/s12936-024-04995-y.

## Background

Malaria continues to pose a serious threat to international health, especially to disadvantaged communities in sub-Saharan Africa [1]. The world's poorest areas, including sub-Saharan Africa, account for almost 90% of all malaria-related morbidity and mortality [1]. The most susceptible age group to malaria is children under five. Nearly every minute, a child under five years of age dies of malaria [2]. A published study shows that sub-Saharan Africa accounted for 80% of malaria-related deaths worldwide in 2021, with children under five being the most affected [1]. The discovery and application of a new malaria vaccine is a promising advance in the fight against malaria. However, implementing a new vaccine in any community is a difficult procedure that needs to take many variables into account. One of the key elements affecting the implementation’s effectiveness is the community’s readiness to embrace and utilize this novel immunization.

It is anticipated that widespread adoption of the World Health Organization (WHO)-approved vaccine, which protects against the *Plasmodium falciparum* malarial parasite, will significantly lower the worldwide burden of malaria-related morbidity and mortality, especially in children [3]. The WHO has reported that 12 sub-Saharan African countries—Ghana, Kenya, Malawi, Benin, Burkina Faso, Burundi, Cameroon, the Democratic Republic of the Congo, Liberia, Niger, Sierra Leone, and Uganda—will now receive 18 million doses of the first-ever malaria vaccine as part of their regular immunization programs over the next two years [2]. The successful implementation of routine malaria immunization programmes signifies a substantial stride forward in combating malaria, which ranks among the leading causes of death in Africa. Since 2019, almost 1.7 million children in Ghana, Kenya, and Malawi have received the RTS, S/ASO1 vaccine, which has been proven to be both safe and effective [2]. As a result, there has been a significant decline in both severe malaria and child fatalities [2]. It is anticipated that the first vaccination doses will reach other countries in the latter half of 2023 and that by early 2024, countries will begin to roll them out [2].

The roll out of the malaria vaccine necessitates a critical examination of “Community readiness and acceptance for the Implementation of this novel vaccine among at-risk children in sub-Saharan Africa.” Previous research, however, has shown that children do not receive enough vaccines, even though services for them are available [4]. This is especially true in low- and middle-income countries, where only approximately 1 in 10 children are fully immunized [5]. Additionally, observational studies in African countries evaluating caregivers’ readiness and acceptance of the malaria vaccine for children under five years have reported contradictory findings [6–9]. Therefore, the purpose of this study is to ascertain the pooled readiness and acceptance of the RTS, S/ASO1 vaccine in sub-Saharan Africa using a systematic review and meta-analysis. Thus, to maximize vaccination coverage and uptake in sub-Saharan Africa, policymakers and other stakeholders may find the current study useful in choosing public health strategies. Additionally, this study will bridge the knowledge gap that currently exists between the development of new medical treatments and their effective application in sub-African Africa.

## Research question

What is the current state of community readiness and acceptance for the implementation of a novel malaria vaccine among at-risk children in sub-Saharan Africa, based on available evidence?

## Methods

### Design

This is a protocol to conduct a systematic review. The review will strictly adhere to the current guidelines for the Preferred Reporting Items for Systematic reviews and Meta-Analyses protocol (PRISMA-P) for 2020 [10]. Additionally, the protocol has been registered with the prospective registers for systematic reviews with a Registration Number CRD42023480528. This protocol will be conducted in five steps.

### Search for literature

Data will be searched using a two-step procedure. Firstly, published studies will be searched from research databases, including PubMed, ScienceDirect, Google Scholar, Web of Science, and African journals online. Secondly, searching for research articles from references of included studies, systematic reviews of a similar topic, and hand searches of unpublished data from institutional libraries and repositories. A primary search string has been developed for the PubMed database (attachment 2) and will be modified for search for the rest of the databases. A broad search will be built using key terms, including all terms and synonyms, Boolean operators, and database-specific thesauri. The key terms include malaria vaccine, readiness, and Africa. The search will not be limited by language and date.

### Eligibility criteria

The inclusion and exclusion criteria has been defined based on the Population, Exposure, Comparator and Outcome (PECO) framework guide [11]. For inclusion, studies must meet the following:

- P: at-risk children (under five years) in Sub-Saharan Africa.
- E: cross-sectional studies assessing the implementation of a novel malaria vaccine.
- C: studies with a comparison group, if available, comparing different levels of readiness or acceptance.
- O: studies on community readiness and acceptance of the novel malaria vaccine.

If any of the included studies have any of the following characteristics, they will be excluded from the analysis:

- Missing on the outcomes that is readiness or acceptance.
- Commentaries or letters to editors.
- Full-texts not accessible for data extraction.

### Study selection

After performing the database search, articles will be exported to Rayyan software in the research information system (RIS) standardized tag format for screening. A four-stage selection process including screening titles and abstracts, retrieving full articles, screening full texts, and selecting full texts will be employed [12]. Initially, studies will be included or excluded based on titles and abstracts. All included studies will undergo full-text screening and selection, performed independently by three reviewers who will be blinded. Any disagreements will be resolved through dialogue or, if necessary, by the principal investigator. The entire process will be documented in a flow diagram [10].

### Data extraction from included studies

A prewritten data extraction form designed in Microsoft Excel (2013) will be developed and pretested on a few studies before use. Two independent reviewers will extract the data separately. In case disagreements occur, they will be resolved by discussion and, if necessary, consultation with a third reviewer. For articles obtained but with missing data, the corresponding authors for those studies will be contacted to provide the needed data. The data to be collected will include the author, title, year of publication, country, sample size, sampling strategy, demographic factors, level of willingness, level of acceptance, and associated factors, among other relevant data.

### Risk of *bias* assessment

The risk of bias for the included studies will be assessed using the Joanna Briggs Institute (JBI) Critical Appraisal Checklist for cross-sectional studies [13]. However, no study will be excluded based on its quality. Studies will be categorized as having high or low quality based on a set of eight questions [14].

### Data management and synthesis

Before data analysis, the data in the extraction sheet will be cleaned and organized in a form that can be read by the analytical software. The degree of observed heterogeneity and feasibility to conduct a meaningful quantitative synthesis will be assessed. If heterogeneity is beyond what can be adequately managed through statistical methods and subgroup analysis, a narrative synthesis will be conducted to provide a summary of the level of community readiness or acceptance for the novel malaria vaccine. In case the data are adequate, a meta-analysis will be performed to estimate the pooled prevalence of readiness or acceptance for all included studies. This will be presented on a forest plot with individual studies included. The effect of each study will be shown as a square box with a horizontal line as a 95% confidence interval, whereas the overall effect (pooled prevalence) will be shown as a diamond, with its length symbolizing the confidence interval. To choose whether to use a fixed-effects or random-effects model, the variability between included studies will first be assessed through the I^2^ statistic. Where high heterogeneity exists (more than 50%), the random-effects model for obtaining the common effect will be used [15]. The index of heterogeneity (I^2^) will be calculated with uncertainty intervals to indicate its level of precision. I^2^ is based on the Q statistic, obtained as the sum of squared deviations of each study’s estimate from the overall estimate. When at least two studies report on a given predictor of readiness or acceptance, odds ratio (OR) with a corresponding 95% confidence interval will be assessed. These will be indicated on the forest plot graph. Each study in the plot will be represented by a square whose size is proportional to effect size (OR) and a horizontal line indicating the 95% confidence interval. The diamond will represent the OR and its length will symbolize the corresponding 95% confidence interval. Where the odds ratio crosses the null value (1.0) indicated by the solid vertical line in the plot will indicate no statistical significance.

To preserve the validity of the review, publication bias will be assessed and, if present, controlled. As a rule of thumb, publication bias is assessed graphically through analysis of funnel plot asymmetry when at least 10 studies are included in the meta-analysis [16]. Asymmetry of the funnel plot will indicate potential publication bias. Additionally, a non-significant p value (p > 0.05) of Egger’s test will indicate the absence of publication bias. Additional subgroup analysis and sensitivity analysis will be performed. Subgroup analysis will be performed based on the expected sources of heterogeneity, such as regions, year or publication, and quality of included studies. If over 10 studies have been included in the analysis, leave-one-out sensitivity analysis will be performed to assess the effect of inclusion of each study on the overall estimate [17].

All the statistical analysis will be performed using STATA version 17 (StataCorp LLC, College Station, TX) with the metan package for pooling effect sizes and conducting overall meta-analyses.

## Discussion

The proposal to assess community readiness for the implementation of a novel malaria vaccine among at-risk children in sub-Saharan Africa is of paramount importance in ongoing global efforts to combat this deadly disease. Malaria remains a significant public health challenge in Sub-Saharan Africa, where children are particularly vulnerable [18]. The introduction of a new vaccine represents a promising opportunity to reduce the burden of malaria, but its success hinges on the readiness and acceptance of the affected communities [19]. This systematic review protocol aims to comprehensively evaluate the literature on community readiness in the context of malaria vaccine implementation, shedding light on factors such as local knowledge, attitudes, and cultural beliefs that can influence the acceptance and uptake of the vaccine. By systematically reviewing this body of work, the proposal intends to provide essential insights into the specific challenges and opportunities in sub-Saharan Africa, thus informing targeted interventions and strategies that can enhance the successful deployment of the novel malaria vaccine, ultimately saving lives and reducing the malaria burden among at-risk children in the region.

Furthermore, the proposal underscores the need for a multifaceted approach that not only considers the effectiveness of the vaccine itself but also takes into account the social, cultural, and infrastructural factors that influence community readiness. It acknowledges the importance of community engagement and empowerment, acknowledging that local stakeholders, including community leaders and healthcare providers, are integral in fostering acceptance and building trust in the vaccine [20]. By synthesizing the available evidence, the proposal seeks to guide policymakers, healthcare professionals, and international organizations in tailoring their interventions to address the unique needs and challenges of communities in sub-Saharan Africa. This systematic review protocol, when carried out comprehensively and rigorously, has the potential to inform evidence-based strategies that will contribute to the successful implementation of a novel malaria vaccine, bringing us one step closer to a malaria-free future for at-risk children in sub-Saharan Africa.

## Conclusion

The study holds the promise of significantly advancing our understanding of the complex dynamics surrounding the introduction of a novel malaria vaccine in a region burdened by this devastating disease.

### Supplementary Information

Supplementary material. 1

## Data Availability

All the data used will be available from the corresponding author upon reasonable request.
