# Supplementary material for: Community readiness and acceptance for the implementation of a novel malaria vaccine among at-risk children in sub-saharan Africa: a systematic review protocol
Source: Malar J. 2024 Jun 10;23:182. doi: 10.1186/s12936-024-04995-y (PMC11165811; doi:10.1186/s12936-024-04995-y)
Supplement: Supplementary file 1 — Supplementary material. 1 [file 12936_2024_4995_MOESM1_ESM.docx]

| PubMed | **Search Terms** | **Records** |
| --- | --- | --- |
| #1 | readiness OR willingness OR acceptance | 613,070 |
| #2 | RTSS OR RTS,S OR RTS,S/AS01 OR (RTSS/AS01 OR “Malaria vaccine” OR Mosquirix OR MVIP OR MVPE OR Immunization | 568,541 |
| #3 | Children OR infant OR infants OR toddler OR toddlers OR kid OR kids OR juvenile OR “children below 5 years” OR minor OR minors OR “young children” OR “under 5 years” OR “under 5” OR “children 0–59 months” | 3,313,008 |
| #4 | “sub-Saharan Africa” OR Africa OR SSA OR Angola OR Benin OR Botswana OR “Burkina Faso” OR Burundi OR Cameroon OR “Cape Verde” OR “Central African Republic” OR Chad OR Comoros OR Congo OR “Cote d'Ivoire” OR Djibouti OR “Equatorial Guinea” OR Ethiopia OR Eswatini OR Gabon OR “The Gambia” OR Ghana OR Guinea OR “Guinea-Bissau” OR Kenya OR Lesotho OR Liberia OR Madagascar OR Malawi OR Mali OR Mauritania OR Mauritius OR Mozambique OR Namibia OR Niger OR Nigeria OR Rwanda OR “Sao Tome and Principe” OR Senegal OR Seychelles OR “Sierra Leone” OR Somalia OR “South Africa” OR Sudan OR Swaziland OR Tanzania OR Togo OR Uganda OR Zaire OR Zambia OR Zimbabwe | 1,080,388 |
| #5 | #1 AND #2 AND #3 AND 4 | 19,383 |
